# The feasibility of the introduction of the Ross program in adults: early safety and quality outcomes in a single center

**DOI:** 10.1007/s12471-026-02062-6

**Published:** 2026-07-29

**Authors:** Nabil Saouti, Anthonie L. Duijnhouwer, Tim J. Ten Cate, Arie P. J. van Dijk, Robin H. Heijmen

**Affiliations:** 1 Department of Cardio-thoracic Surgery, Radboud University Medical Center, Nijmegen, The Netherlands; 2 Department of Cardiology, Radboud University Medical Center, Nijmegen, The Netherlands

**Keywords:** Short-term outcomes, Aortic valve, Homograft, Aortic valve regurgitation, Aortic valve stenosis

## Abstract

**Background:**

The optimal valve substitute for young and middle-aged patients with severe symptomatic aortic valve disease remains a subject of debate. In this patient group, prosthetic valves are associated with reduced survival, impaired hemodynamics, and lifelong risk of valve-related complications. The Ross procedure has re-emerged as the most optimal valve substitute following surgical refinements and improved perioperative care. The current study describes the feasibility in terms of safety and early outcomes of a newly initiated Ross program using the patient-tailored total root technique.

**Methods:**

All patients who underwent the Ross procedure at the Radboud University Medical Center between April 2024 and December 2025 were included. Baseline characteristics, operative details, and postoperative outcomes were extracted from the electronic medical record.

**Results:**

A total of 32 patients were included, with a median age of 39 years; 65% were male, and 94% had a bicuspid aortic valve. The predominant pathology was pure stenosis in 63%. All procedures were elective except one. There was no operative or 30-day mortality. Complications included one conversion to a Bentall procedure for leaflet retraction and one urgent percutaneous coronary intervention, probably due to right coronary button kinking. No patient had more than mild aortic regurgitation at discharge.

**Conclusions:**

The Ross procedure in young and middle-aged adults was associated with favorable short-term outcomes in this initial cohort. These results support the feasibility and safety of the Ross procedure when performed in a dedicated expert center. Ongoing follow-up will be essential to assess medium- and long-term durability.

## Introduction

The optimal valve substitute for young and middle-aged patients with severe symptomatic aortic valve disease (stenosis and/or regurgitation) remains a subject of debate. Balancing the potential risks and benefits poses a dilemma for both physicians and patients.

According to the 2025 ESC/EACTS guidelines, valve replacement with a mechanical prosthesis should be preferred or considered (Class 2a, C) for patients under 60 years of age [1]. However, in young and middle-aged patients, mechanical prostheses, and even more so biological prostheses, have been associated with reduced survival and life-long risk of major valve-related complications (i.e., mechanical valves necessitate lifelong anticoagulation with bleeding/thrombo-embolism risk, while bioprostheses have limited durability with higher risk of reintervention) [2, 3].

The Ross procedure, involving translocation of the pulmonary autograft, was introduced in the 1960s with the concept that a ‘living’ aortic valve substitute can normalize hemodynamics, restore life expectancy, and improve quality of life [4]. It subsequently gained popularity and widespread use. However, interest then declined due to higher operative risk, including operative mortality, autograft failure, and early reoperations compared with standard aortic valve replacement. However, major refinements in surgical technique and perioperative care have generated growing evidence from dedicated centers indicating that the Ross procedure is the most favorable valve substitute for non-elderly patients with aortic stenosis and/or regurgitation, leading to renewed interest. Contemporary data consistently demonstrate restored survival up to the third decade after Ross surgery, normal hemodynamics at rest and during exercise, low rates of valve-related complications, uncomplicated pregnancies, and excellent quality of life [5, 6].

At our center, with extensive surgical experience in complex aortic root procedures such as Bentall operations (including redos) and valve-sparing root replacements, we recently reintroduced the Ross procedure for young and middle-aged adults with severe aortic stenosis or non-repairable aortic regurgitation. The aim of this study is to evaluate the short-term outcomes of our newly initiated Ross program.

## Methods

All patients who underwent the Ross procedure since the start of the program in April 2024 up to and including December 2025, at the Radboud University Medical Center were included.

### Patient selection

At our institution, the Ross procedure is considered a viable option for adult patient aged 18 to approximately 55–60 years with aortic valve stenosis or non-repairable aortic regurgitation, depending on lifestyle, activity level, and life expectancy. Each patient is discussed and approved by the heart team for surgical aortic valve replacement according to current international guidelines. Additional preoperative work-up for the Ross procedure includes computed tomography imaging of the thoracic aorta and coronary arteries. The presence of anomalous coronary arteries or suspicion of calcific stenosis warrants coronary angiography for complete assessment.

Once the patient consents to the Ross procedure, a pulmonary homograft is ordered. Oversized homografts (> ~27 mm) are selected to optimize hemodynamics and to mitigate the risk of future homograft stenosis, which may occur as a result of an inflammatory response [7, 8].

Contraindications for the Ross procedure include rare coronary anomalies, such as a right coronary artery originating from the left anterior descending artery, large pulmonary valve fenestrations (which can only be assessed intraoperatively), patients engaged in high-intensity isometric exercise (e.g., competitive weightlifting), connective tissue disease, and the active phase of rheumatic or autoimmune disorders.

### Surgical technique

We adopted the total root technique as described by Williams and El-Hamamsy [9]. In short, the approach is through a median sternotomy. The autograft was implanted with single interrupted Prolene sutures in the subannular plane of the native left ventricular outflow tract. In patients with predominant aortic regurgitation, usually associated with a dilated aortic annulus, an extra-aortic annuloplasty (Dacron ring or Coroneo ring, Coroneo Inc., Montreal, Canada) was placed at the level of the basal ring. Subsequently, the coronary buttons were implanted in the sinus body of the autograft. In case of a dilated native ascending aorta (> 38 mm), a Dacron graft was interposed; otherwise, the autograft was directly sutured to the native ascending aorta.

The remnant tissue of the non-coronary sinus and residual commissural tissue between the native right and left sinuses were fixed with sutures to the adventitia of the native aorta, or on the Dacron graft if used (so-called ‘loose jacket technique’) to prevent autograft dilatation.

Since pulmonary homografts are scarce, the (largest) Freestyle stentless porcine root prosthesis (Medtronic Inc., Minneapolis, MN, USA) was used as an alternative in relatively older patients (generally > 45 years) [10].

All patients underwent transthoracic echocardiographic evaluation at our hospital before discharge or transfer to another facility.

### Postoperative care

Strict blood pressure control, aiming at systolic blood pressure below 110–115 mm Hg, immediately after surgery and maintained for at least 6 months to 1 year, is of particular importance to prevent early autograft dilatation. A specialized nurse maintained weekly contact with each patient to monitor home blood pressure measurements and adjust therapy according to a strict protocol.

### Definitions and data analysis

Baseline variables, operative details, and postoperative outcomes were extracted from the electronic medical record using a standardized template. Operative mortality was defined as death during the index hospitalization or within 30 days after surgery. Reoperation was defined as any unplanned return to the operating room during the index admission. Reintervention included any unplanned percutaneous or surgical procedure related to the aortic or pulmonary position. Conduction disturbance was defined as the need for a new permanent pacemaker. Myocardial infarction was defined by clinical symptoms and biomarker elevation with corroborating findings on electrocardiography. Stroke was defined as a new neurological deficit persisting longer than 24 h. Aortic regurgitation severity was graded on transthoracic echocardiography according to current guidelines and reported as none/trace, mild, moderate, or severe at discharge.

Continuous variables are reported as median with interquartile range or as mean with standard deviation; categorical variables as counts and percentages. All data were analyzed descriptively. Operative variables were analyzed reflecting efficiency endpoints. Post-operative variables were analyzed reflecting safety and efficacy endpoints.

## Results

In total, 32 patients underwent a Ross procedure since the program restarted in April 2024.

Mean follow-up was 9.6 ± 4.2 months. In Tab. 1, our total case load of proximal aortic valve and root surgery, including redo procedures, is outlined.

**Table 1:** Total case load aortic valve/root program 2024–2025

*AVR (redo) ≤55 years of age*	*29 (17)*
*Bentall (redo)*	88 (12)
*Aortic valve repair*	8
*Aortic valve sparing root replacement (redo)*	33 (1)

Total case load of proximal aortic valve/root practice in a single-center in 2024 and 2025

Baseline characteristics are presented in Tab. 2. The median age was 39 years (interquartile range 22), and 63% of patients were male. Two patients had previous cardiac surgery in childhood. The majority of patients had a bicuspid aortic valve (94%), and pure stenosis was the predominant valve dysfunction in 63%. All patients underwent elective surgery, except for one (case 9), who was hospitalized with syncope due to complete atrioventricular block. A temporary transvenous pacing wire was placed as a bridge to surgery. During the Ross procedure, permanent epicardial atrial and ventricular pacing leads were placed in preparation for a permanent pacemaker after surgery.

**Table 2:** Baseline characteristics (*n* = 32)

Characteristic	Value
Age at surgery, mean ± SD	39 ± 12
Median (IQR)	39 (22)
18–25 yrs, *n*	6 (19%)
26–35 yrs, *n*	8 (25%)
36–45 yrs, *n*	6 (19%)
46–55 yrs, *n*	12 (37%)
Gender, male (%)	21 (65%)
Body mass index, mean ± SD	28 ± 4
Diabetes mellitus	1 (4%)
Chronic obstructive pulmonary disease	1 (4%)
Peripheral artery disease	0
Previous CVA or TIA	0
Previous cardiac surgery	2 (8%)
Previous cardiac intervention	3 (12%)
*Preoperative rhythm*	
– Atrial fibrillation	3 (9%)
– pacemaker	1 (4%)
*Left ventricular function*	
– > 50%	23 (72%)
– 31–50%	9 (28%)
*Aortic valve morphology*	
– tricuspid	2 (6%)
– bicuspid	30 (94%)
*Valve dysfunction*	
– stenosis	20 (63%)
– regurgitation	5 (16%)
– combined, predominant stenosis	6 (19%)
– combined, predominant regurgitation	1 (3%)
*CT measurement*	
– root/sinus (mm), mean ± SD	36.5 ± 5.8
– ascending aorta (mm), mean ± SD	39.4 ± 7.0

Baseline characteristics of 32 patients undergoing the Ross procedure in a single-center between April 2024 and December 2025

Tab. 3 presents intraoperative variables depicting efficiency endpoints. Ascending aorta replacement with a Dacron graft was performed in half of the patients (47%), and an extra-aortic annuloplasty was applied in 5 patients (16%).

**Table 3:** Efficiency endpoints (*n* = 32)

Characteristic	Value
Cardiopulmonary bypass time [min], median (IQR)	228 (48)
Aortic cross clamp time [min], median (IQR)	176 (41)
Hospital length of stay [days], median (IQR)	7 (3)
*Concomitant procedures*	
– rhythm surgery, *n*(%)	2 (6%)
Ascending replacement, *n*(%)	15 (47%)
Freestyle prosthesis as RVOT substitute, *n*(%)	8 (25%)
*Extra-aortic annuloplasty*	
– dacron ring, *n*(%)	2 (6%)
– coroneo ring, *n*(%)	3 (9%)

Operative characteristics reflecting efficiency endpoints of 32 patients undergoing the Ross procedure before hospital discharge

Short-term outcomes, including complications as safety endpoints and pre-discharge echocardiographic findings as efficacy endpoints, are presented in Tab. 4. No operative and no 30-day mortality was observed. One patient (male, 54 years) had a deep sternal wound infection one month after surgery and could be refixed with Redon drains. After 6 weeks of antibiotics, he had fully recovered.

**Table 4:** Safety and efficacy endpoints (*n* = 32)

Characteristic	Value
Major complications,* *n*(%)	
Mortality, *n*(%)	0
– perioperative	0
– intraoperative	0
Prolonged intubation (> 24 h), *n*(%)	1 (3%)
Perioperative myocardial infarction, *n*(%)	1 (3%)
Conversion to Bentall procedure	1 (3%)
New temporary dialysis, *n*(%)	0
Acute renal failure without need of dialysis, *n*(%)	0
Cerebrovascular accident, *n*(%)	0
Transient ischemic attack, *n*(%)	0
Delirium, *n*(%)	1 (3%)
New pacemaker implantation, *n*(%)	0
Deep sternal wound infection, *n*(%)	1 (3%)
*Predischarge transthoracic echocardiography*	
– aortic regurgitation, no/trace/mild, (*n*)	3/22/7
– aortic gradient, mean ± SD (mm Hg)	3.9 ± 2.3
– pulmonary regurgitation, no/trace/mild, (*n*)	13/17/2
– pulmonary gradient, mean ± SD (mm Hg)	8.4 ± 4.3

Short-term outcomes and echocardiographic outcomes reflecting safety- and efficacy endpoints respectively, of 32 patients undergoing the Ross procedure

Importantly, no patient had more than mild aortic regurgitation on the pre-discharge echocardiography.

Case 17 was a 53-year-old male who underwent a Ross procedure for aortic stenosis with a bicuspid valve. After the release of the cross-clamp, moderate eccentric aortic regurgitation persisted. Following a second cross-clamp and inspection of the autograft, a retracted right leaflet was identified. As the mechanism and potential repair strategy remained unclear, conversion to a mechanical Bentall procedure was performed. The postoperative course was uneventful, with 1 day in the Intensive Care Unit (ICU) and discharge home after 1 week.

Case 19 was a 53-year-old male who underwent a Ross procedure for aortic stenosis with a bicuspid valve. The right coronary ostium was displaced, and the right button was reimplanted similarly on the autograft. The operation was uneventful, with easy separation from cardiopulmonary bypass and good biventricular function on transesophageal echocardiography, as well as good autograft function. However, within 1 h in the ICU, the patient developed ischemic changes on the electrocardiogram and hemodynamic deterioration. Emergency coronary angiography revealed a pinpoint stenosis of the dominant right coronary ostium, likely due to kinking of the button, and immediate percutaneous coronary intervention with a drug-eluting stent was performed. After 2 weeks of recovery in the ICU, he was transferred to the ward, and the rehabilitation was otherwise uneventful. The patient is almost 6 months post-surgery and has fully resumed normal daily activities. Recent follow-up transthoracic echocardiography showed normal left ventricular function without wall motion abnormalities and improved, though slightly reduced, right ventricular function.

## Discussion

The Ross operation is increasingly regarded as the optimal valve substitute for children, and for young and middle-aged adults [3, 6, 11]. In the present cohort, the Ross procedure in young and middle-aged patients was associated with very low operative risk and excellent short-term clinical and echocardiographic outcomes.

The pulmonary autograft is a living valve that is a mirror image of a normal aortic valve and the only aortic valve substitute that preserves the aortic root as a functional unit, so-called *root dynamism*, with long-term viability that translates into clinically important endpoints in both the short and long term [12]. The Ross procedure is particularly advantageous for patients with small aortic annuli, given its hemodynamic benefits that remain stable over time and the absence of patient-prosthesis mismatch [13].

In contrast, prosthetic valves, both mechanical and biological, particularly in patients under 60 years, are associated with reduced survival. This is likely due to inferior hemodynamics with higher gradients that tend to increase over time, especially in highly active patients. These prosthetic valves are non-living and lack the ability to adapt, repair, or remodel. Additionally, they have a rigid sewing ring that immobilizes the LVOT/annulus, preventing annular expansion and torsion of the LVOT during the cardiac cycle, especially with exercise. Moreover, reduced survival is also likely due to the lifelong risk of major valve-related complications [3, 14, 15].

**Fig. 1:**
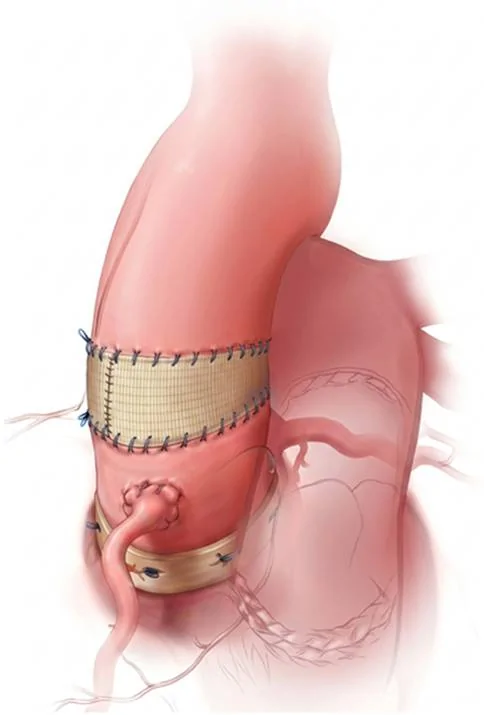
Ross procedure technique; Full root replacement technique. Reprinted with permission from Mazine et al. (2018) [17]

Due to past concern of the potential long-term failure of 2 valves [16] the Ross procedure has evolved over time with major technical modifications, from subcoronary to total root replacement (Fig. 1), and refinements to address late neo-aortic root dilatation causing insufficiency. These refinements constitute a patient-tailored approach and include: (1) prevention of aortic annulus dilatation by inserting the autograft subannular and, in cases of annular dilatation and/or aortic/pulmonary mismatch, performing an extra-aortic annuloplasty; (2) stabilization of the neo-aortic sinus using the loose jacket technique, the autologous inclusion technique, or the Dacron wrapping technique; and (3) stabilization of the neo-sinotubular junction when the native ascending aorta is dilated, using an interposition Dacron graft [17].

One of the main reasons for the decline in use of the Ross procedure was the concern about increased operative mortality. A study by Reece et al. reported a higher operative mortality for the Ross procedure compared with conventional AVR [18]. However, closer inspection of the data showed that most of the 231 centers included in this study performed less than 1 Ross procedure per year, and only 6 centers performed 5 or more procedures annually. As with many complex procedures, a clear relationship has been demonstrated between institutional volume and operative outcomes in aortic root surgery [19, 20]. In contemporary practice, the perioperative mortality associated with the Ross procedure is well below 1% and comparable to conventional AVR, albeit in reference centers [21–23]. These data emphasize that excellent long-term outcomes can be achieved with low operative risk comparable to conventional AVR, provided the procedure is performed in reference centers. This evidence provides the rationale for reintroducing the Ross procedure for adolescent and middle-aged patients. With prior existing experience in root surgery and mentorship from an experienced Ross surgeon, similarly excellent early hospital outcomes were reproduced in the present cohort.

Another concern with the Ross procedure is late failure of the autograft and/or the homograft [16]. Several adjunctive techniques have been developed to minimize the risk of autograft dilatation and regurgitation. Techniques to be effective in this regard include the total inclusion technique (i.e., incorporating the autograft within the fully preserved native root) [24], implantation of the autograft within a Dacron graft [25, 26], autograft support with Personal External Aortic Root Support (PEARS) [27], and the loose jacket technique [28]. In the current cohort, the loose jacket technique was applied, as this method best preserves root dynamism, which, together with the fact that the pulmonary autograft is a living valve substitute, is likely a key determinant of the excellent long-term durability and outcomes of the Ross procedure [12].

The risk of late pulmonary homograft failure, primarily due to valve stenosis, can be mitigated by using an oversized homograft. In the Netherlands, homografts are not commercially available but allocated by the Dutch Transplantation Foundation (NTS). Because of the scarcity of pulmonary homografts, the Freestyle stentless porcine aortic root prosthesis (Medtronic Inc., Minneapolis, MN, USA) was used as an alternative in older patients. Recent data have shown that its durability is comparable to that of homografts for up to 15 years after implantation [10].

The Ross procedure has been offered to all eligible adult patients, also in the current study, irrespective of valve etiology (stenosis and/or regurgitation) and valve morphology (tricuspid, bicuspid, or unicuspid). Usually, in patients with pure aortic valve regurgitation, with or without root dilatation, valve repair is generally preferred. However, when the valve is unrepairable or unlikely to be durably repairable, the Ross procedure represents an excellent alternative. Although the presence of regurgitation is an independent predictor of autograft reintervention after the Ross procedure, studies have shown that, despite late auto- or homograft reintervention, survival remains comparable to that of patients operated on for aortic valve stenosis [6, 29, 30]. To mitigate the increased risk of autograft failure and aortic regurgitation, a patient-tailored approach has been described by the group of El-Hamamsy [31] and was applied in the current cohort. This approach specifically addresses dilatation of the aortic annulus, mismatch between the aortic and pulmonary annuli, and ascending aortic dilatation. This approach has shown comparable outcomes with respect to freedom from recurrent aortic regurgitation and autograft failure [32].

Despite the excellent long-term results and low perioperative risk, the complexity of the Ross procedure, with its numerous potential pitfalls, has limited widespread adoption. As with many complex surgical procedures, the Ross procedure can be reproduced, provided there is extensive experience with root surgery and close mentorship from an experienced Ross surgeon.

In discussions with patients, it is essential to place the Ross procedure in perspective to support shared decision-making. Patients must be informed that the potential long-term advantages of the Ross procedure (excellent hemodynamics, normalization of life expectancy, absence of anti-coagulation, and very low valve-related complications) should be weighed against the complexity of the operation and the associated higher operative risk. However, these operative risks can be minimized to levels comparable with standard surgical AVR when the procedure is performed in dedicated reference centers. Therefore, as endorsed by the latest expert consensus, it is of paramount importance that this surgery is performed in so-called Ross Centres of Excellence, ideally performing 20 to 25 procedures per year [33].

## Conclusion

Short-term outcomes of the Ross procedure in this cohort of young and middle-aged adults are very promising. We believe extensive experience in aortic root surgery is a prerequisite and that maintaining sufficient procedural volume is essential to achieve excellent results. Although these short-term outcomes are encouraging, medium- and long-term results must be closely monitored.

## Data Availability

All data supporting the findings of this work are included in the article.
